# 41. Localised amyloid: a rare cause of stridor

**DOI:** 10.1093/rap/rky034.004

**Published:** 2018-09-20

**Authors:** Klara Morsley, Khin Yein, David Collins, Sara Carty, Elizabeth Price, Rosemary Waller, Azeem Ahmed

**Affiliations:** Rheumatology, Great Western Hospital, Swindon, UNITED KINGDOM

**Introduction:** We present a case of a 68 year old female who was admitted with stridor, and subsequently diagnosed with localised subglottic amyloid. The case highlights the importance of keeping an open mind to a wide differential to ensure that rare causes are not missed.

**Case description:** A 68 year old female patient presented with stridor requiring an emergency tracheostomy. She had a preceding history of two years of a hoarse voice and had recently had a microlaryngoscopy and biopsy of a granuloma on the left vocal cord. Further examinations of the subglottic area revealed oedema with friable areas, and the subglottic biopsy showed amyloid deposition. She was systemically well, with no symptoms elsewhere. She was investigated for causes of secondary amyloid but none found. Her inflammatory markers were normal, ANCA, ANA and anti-CCP were negative with normal serum ACE and complement levels. Serum electrophoresis and bone marrow biopsy were both normal. She was started on a reducing regimen of prednisolone with clinical improvement and successfully extubated. After discharge, she went on to have a SAP scan which showed the amyloid to be localised to the subglottic area, and a subsequent CT-PET showed the biopsy proven amyloid area to be markedly FDG avid with disease extending inferiorly along the trachea Over the next month, she developed increasing shortness of breath on exertion and stridor, requiring up to 40mg prednisolone, and has been referred for consideration of surgical intervention.

**Discussion:** Localised amyloid is a rare disease. In a study at the National Amyloid Centre between 1980 and 2011, 12% of all newly diagnosed patients with amyloid had localised disease with a median age was 59.5 years. 15% of these patients had amyloid localised to the larynx or tonsils. Primary laryngeal amyloid is seen more often within the laryngeal ventricle or vocal cords with subglottic involvement being less common. It presents with dysphonia and stridor, and as in our patient, may result in airway compromise. Our patient was initially referred to rheumatology with a possible diagnosis of subglottic granulomatosis with polyangiitis (GPA), which may also be localised to the subglottic area and present with similar symptoms. ANCA may also be negative in patients with limited GPA. Consideration of localised amyloid as an alternative is important and Congo Red staining on biopsy will confirm the diagnosis. The prognosis of localised amyloid is generally good, with an estimated five-year overall survival of 90·6% in the National Amyloid Centre study. Although the literature suggests that steroids are not of benefit in localised laryngeal amyloid, our patient appeared to gain clinical benefit from them. The mainstay of treatment is carbon dioxide laser excision, which may need to be repeated.

**Key Learning Points:** Subglottic stenosis may be an airway threatening problem. Localised amyloid should be included in the differential diagnosis for these patients and Congo Red staining performed on biopsy specimens. All patients with localised amyloid should be carefully evaluated for systemic amyloid, and causes of secondary amyloid.

**Disclosure: K. Morsley:** None. **K. Yein:** None. **D. Collins:** None. **S. Carty:** None. **E. Price:** None. **R. Waller:** None. **A. Ahmed:** None.

